# The sounds of safety: stress and danger in music perception

**DOI:** 10.3389/fpsyg.2015.01140

**Published:** 2015-08-05

**Authors:** Thomas Schäfer, David Huron, Daniel Shanahan, Peter Sedlmeier

**Affiliations:** ^1^Department of Psychology, Chemnitz University of Technology, Chemnitz, Germany; ^2^Cognitive and Systematic Musicology Laboratory, Ohio State University, Columbus, OH, USA; ^3^School of Music, Louisiana State University, Baton Rouge, LA, USA

**Keywords:** music, safety, stress, information density, sound

## Abstract

As with any sensory input, music might be expected to incorporate the processing of information about the safety of the environment. Little research has been done on how such processing has evolved and how different kinds of sounds may affect the experience of certain environments. In this article, we investigate if music, as a form of auditory information, can trigger the experience of safety. We hypothesized that (1) there should be an optimal, subjectively preferred degree of information density of musical sounds, at which safety-related information can be processed optimally; (2) any deviation from the optimum, that is, both higher and lower levels of information density, should elicit experiences of higher stress and danger; and (3) in general, sonic scenarios with music should reduce experiences of stress and danger more than other scenarios. In Experiment 1, the information density of short music-like rhythmic stimuli was manipulated via their tempo. In an initial session, listeners adjusted the tempo of the stimuli to what they deemed an appropriate tempo. In an ensuing session, the same listeners judged their experienced stress and danger in response to the same stimuli, as well as stimuli exhibiting tempo variants. Results are consistent with the existence of an optimum information density for a given rhythm; the preferred tempo decreased for increasingly complex rhythms. The hypothesis that any deviation from the optimum would lead to experiences of higher stress and danger was only partly fit by the data. In Experiment 2, listeners should indicate their experience of stress and danger in response to different sonic scenarios: music, natural sounds, and silence. As expected, the music scenarios were associated with lowest stress and danger whereas both natural sounds and silence resulted in higher stress and danger. Overall, the results largely fit the hypothesis that music seemingly carries safety-related information about the environment.

## Introduction

The main purpose of any sensory system is to provide information about the environment, which is then used to accomplish adaptive behavior. Some forms of information are more important than others. For example, information related to both food and reproduction is essential. However, probably the most important category of information relates to safety. Vision and hearing are the two sensory channels that are most important to gather safety-related information. Yet, in comparison with vision, auditory processing of safety-related information appears to be superior because the sense of hearing (1) exhibits a 360° range of detection, (2) is tolerant of obstruction, (3) has a distant event horizon, (4) is neuronally processed very quickly, (5) is inextricably linked with attention and emotion, and (6) is active even while we sleep (see Horowitz, 2012). Surprisingly little research has been done on how the processing of safety-related auditory input has evolved and how different kinds of sounds may affect the experience of certain environments. Cross and Watson (2006, p. 107) have noted that “the role and effect of sound and the human experience of sound in archeological environments is severely under-researched. Sound is a primary source of information about the world, and the human experience of sound shapes many of the ways in which we interact with the world and with each other.” Studies have shown that speed, rhythm, pitch, pitch range, and intensity of sound stimuli can carry information regarding danger (e.g., Rosenblum et al., 1993; Bach et al., 2009; Seifritz et al., 2015) or urgency (Edworthy et al., 1991). There should have been a benefit for individuals when they managed to process auditory input and extract the information effectively. Auditory information catches immediate attention, indicates danger, or signals a stable and safe environment (see Huron, 2006). In primeval natural environments, our ancestors must have needed to rely on external stimuli that signaled safety. Periods of safety in unchallenging places were crucial for them to relax, conserve energy, and sleep. If the auditory input over time provides constant or highly similar information about the environment, then this can be interpreted as an indication of a stable situation that is free from danger and under control. As a result, an individual in such situation would experience reduced stress and no anxiety and maintain a positive affect (see Chorpita and Barlow, 1998).

Can music—as a form of auditory information—trigger the experience of safety? That is, could music be understood as a means to create the illusion of a safe environment where there is no danger, no need to be afraid or alert, and no need to act? Musical sounds are relatively predictable rhythmic patterns that humans might have been produced by their own voice or by simple things such as hollow wood even thousands of years ago (sophisticated instruments came later). Slight variation of these sounds might act as an auditory source of input to our information processing system, which signals the environment is not changing uncontrollably or unpredictably: The incoming stimuli are variable enough to provide an interesting source of information about the environment, but they are also structured and predictable enough to indicate that everything is under control. Some touches of pre–musical behavior—for example, knocking rhythmically on wood, humming—might have been reinforced at some time in human evolution by positive affect. It can be hypothesized that even today we prefer music that is in the range described above: not too predictable, not too variable. Music that is too monotonous or repetitive contains little relevant information; it hardly rouses our interest and is likely to make us bored. Too complex, long–phrased, or unpredictable music does not produce pleasure, either (Berlyne, 1971, 1974; Heyduk, 1975; Kellaris, 1992). Instead, such music increases our arousal and elicits negative affect, because it can be deemed a potential indicator of a dangerous environment (Huron, 2006).

Of course, in creating musical works, a large number of factors are inevitably involved, including social, cultural, political and other aspects. Nevertheless, one might expect that some basic elements of auditory processing when listening to music may originate in perceptions of relative safety or danger (see also Weiss, 1970; Orsini et al., 2002; Herry et al., 2007; Whalen, 2007). If music is indeed at least partly processed as a source of auditory information about the environment we might expect a relationship between music processing and experiences of safety and stress. Two experiments were designed to investigate this general conjecture.

In Experiment 1, we created and systematically manipulated rhythmic sound stimuli in order to vary their information density. We predicted that there is an optimum information density, where each individual experiences lowest stress and danger; both higher and lower information density should increase the experience of stress and danger. Specifically, when sound patterns exhibit a very high degree of information density (for instance, because of an especially fast event rate) there is the danger that a listener may miss certain sounds that are informative pertaining to safety. Accordingly, the listener may experience more stress and danger. On the other hand, sound patterns that exhibit a very low degree of information density (for instance, because they are especially slow) might cause the experience of stress and danger as well because the listener is waiting for input that is not coming (comparable to silence; see below). Hence, the experience of stress and danger should be lowest when the rhythmic stimuli exhibit information density appropriate for the individuals’ level of information processing speed. We dubbed this the *information density* hypothesis.

In Experiment 2, real music was used and contrasted to other types of auditory input that might have been relevant in prehistoric environments, namely natural sounds and silence. We predicted that situations with music are experienced as less stressful and dangerous than situations with silence or natural sounds. This hypothesis may sound counterintuitive since many would expect that silence was the most reliable indicator of safety. What follows from our above arguments, however, is that silence should produce an experience of unease because it simply does not contain any information at all. It was therefore hard or impossible for an individual to identify whether a situation was safe or not. We would therefore expect that a silent environment is not experienced as less stressful or dangerous than a situation with a natural acoustic background (i.e., nature sounds). By contrast, music—representing a relatively predictable pattern of auditory input—should create the impression that nothing dangerous is going on in the environment.

## Experiment 1—The Relationship Between Information Density of Rhythmic Sound Stimuli and the Experience of Stress and Danger

The purpose of Experiment 1 was to manipulate the information density of simple rhythms unknown to participants and then measure what experiences these rhythms elicit. If our hypothesis is correct there should be an optimum level of information density of auditory information with which an individual feels most safe with—both more complex and less complex stimuli should lead to an increase in experienced stress and danger. Information density was to be manipulated around an optimum point, that is, a degree that a specific listener experiences as appropriate. There are many possibilities to manipulate information density but we set out to create stimuli that are manipulated along only one single dimension, and have listeners identify the optimum point along this dimension. For the purposes of this study, we elected to manipulate the tempo. Our conjecture was that there is an optimum tempo for any given music–like stimulus, and that this optimum is related to the experience of highest safety and lowest stress. That is, the optimum tempo will be associated with the least threatening information flux. Specifically, we hypothesized that there is an optimum tempo of played rhythms for each listener, depending on the individual cognitive processing capacity and processing speed, and that the rhythms of optimum tempo are experienced as least stressful and most safe.

### Materials and Methods

The experiment consisted of two parts separated by at least 1 week. In the first part, participants listened to a series of nine rhythms and were asked to modify the tempo to achieve what they thought was a suitable speed. In the second part, participants rated their experienced degree of stress and safety in response to the rhythms of Part 1—including both manipulated and unmanipulated versions. The first session was approximately 30 min in duration and the second session was approximately 60 min.

#### Sample

In total, 37 undergraduates completed both parts of the experiment (mean age: *M* = 22.7 years; SD = 4.5; 28 females, 9 males). They all reported unimpaired hearing by a self-rating. The study was performed in accordance with relevant institutional and national guidelines and regulations (Chemnitz University of Technology, 2002; Deutsche Gesellschaft für Psychologie [German Psychological Society], 2005). Informed consent was obtained from all participants. Anonymity of participants and confidentiality of their data were ensured.

#### Stimuli and Materials

To minimize the effect of style and cultural presuppositions, we aimed to create music-like stimuli that were not too similar to or reminiscent of Western music. Accordingly, we made use of a simple Midi instrument that employed the percussive patches from the General MIDI bank of sounds (specifically, mid-tom, and high-tom sounds). The rhythms were all in 5/4 meter, which is relatively infrequent in Western music. Two versions were used: 3+2 and 2+3 groupings. Metric hierarchies were created for each of these metric types, and rhythms were generated from these hierarchical distributions. To ensure a wide range of rhythms, the onset densities varied from as few as two onsets per measure to as many as 20 onsets per measure. Using information-theoretic measures, it is possible to characterize the information density (complexity) of each rhythm in bits (see below).

Although all of the rhythms were constructed in 5/4 meter, the beat subdivisions were based on statistics gathered for Western rhythms. We used the metric hierarchy for a sample of Western music in 4/4 meter. Specifically, this distribution was assembled from a set of 45 common American songs and 164 traditional German folk songs (Schaffrath, 1995), all in 4/4 meter. The distribution represents the typical hierarchy where beats 1 and 3 are most important, followed by beats 4 and 2. An analogous hierarchy is evident at the sub-beat level. Accordingly, we generated our rhythms using these same distributions—modified to a 2+3 or 3+2 hierarchy. In 4/4 meter, the first beat is the strongest (“S”) and the third beat second strongest (“s”); the second beat is weakest (“w”) and the fourth beat is considered a pickup or anacrusis (“a”) to the ensuing downbeat. In creating the metric hierarchies for the 5/4 meter we retained the analogous functions. For example, the 3+2/4 meter would exhibit an S–w–w–s–a pattern, whereas the 2+3/4 meter would exhibit an S–w–s–w–a pattern. Using the novel metric templates, a large number of rhythms were generated using a random procedure based on the simple zeroth-order probabilities. Because first- and higher order probabilities play no role in the generation of these rhythms, many of these rhythms are apt to be regarded as quite complex. Apart from the zeroth-order distribution of events within a metric hierarchy, rhythms commonly exhibit significant first-order constraints. For example, in 4/4 meter, an onset coinciding with beat 2 significantly raises the probability of an ensuing onset coinciding with beat 3. Moreover, if an event were to occur on beat 2, without an ensuing event on beat 3, the effect would be a marked syncopation (see Huron, 2006, Chapter 10). Accordingly, we might make use of the first-order probabilities as a convenient way of measuring the information density of the ensuing rhythms.

We sought to create some variance in the information density of the rhythms. The information density of each resulting rhythm was characterized using the Shannon–Weaver equation (Shannon and Weaver, 1963). From a random sample of 10,000 generated rhythms, the rhythms were categorized into three conceptual categories (corresponding to simple, moderate, and complex rhythms): high (> 10 bits), medium (5–10 bits), and low (< 5 bits) information density. For the purposes of the experiment, an equivalent number of rhythms were selected from these three categories. Rhythmic stimuli were generated uniquely for each participant. That is, to maximize data independence, no rhythm was used for more than one participant. Nine stimuli were generated for each participant, three each in the high, medium, and low information density categories. As a result, there were a total of 333 rhythmic stimuli, all of which were generated using MaxMSP.

In generating our stimuli, several questions arose, including what the duration of the stimuli and the amount (if any) of internal repetition (such as repeated measures) should be. Repetition is known to increase a participant’s preference for stimuli. This phenomenon is variously known as the mere exposure effect (Zajonc, 1968), processing fluency (Bornstein, 1989), or the prediction effect (Huron, 2006). To minimize the effect of repetition, our stimuli were generated without repetition. One approach to minimizing repetition is to employ short stimuli, such as a single bar of 5/4. Such stimuli would be roughly 2–3 s in length. It is plausible, however, that our participants would be unable to form robust impressions of such short stimuli. Consequently, we aimed for stimuli in the range of 30 s in duration. For rhythms in 5/4 meter, at a moderate tempo, this amounts to some 12 measures. Once again, to minimize the effect of repetition on liking, we chose to avoid repeated rhythms within the 30-s stimulus duration. That is, rather than generate a single 5/4 bar and repeating this rhythm, each bar contained a unique rhythmic pattern. To facilitate perception of the meter, we mapped downbeats (first beat in the measure) to a high pitch on the Midi instrument and other beats to a lower pitch.

We expected the perception and optimum processing of rhythmic stimuli to depend on the listener’s cognitive processing capacity and speed. Therefore, we asked participants to complete a 3-back test (a version of the *n*-back test; e.g., Gevins and Cutillo, 1993) to measure cognitive processing capacity and the Trail-Making Test (see Giovagnoli, 1996) to measure processing speed. For the 3-back test, the relative number of correct answers were measured. For the Trail-Making Test, the speed was measured.

#### Procedure

***Session 1***

After providing informed consent, participants were seated in front of a computer screen in a silent and darkened lab room. All instructions were given on the screen. Participants wore headphones and were asked to imagine they were sitting at a campfire somewhere in the savanna, and listen to different rhythms. They could close their eyes if they wanted to. In the first session, participants heard the individual rhythmic stimuli and used a method–of–adjustment procedure to tune an optimum tempo. Participants received the following instructions:

In this experiment, you’ll be presented with a sequence of 9 rhythms that will sound rather unfamiliar to you as they are composed in 5/4 meter. They are based on rhythms that are still common in some regions in Africa. So just imagine you are sitting at a campfire in the savanna, listening to these rhythms. You can close your eyes if you like. Each rhythm will repeat continuously. You can start or stop the sound by clicking on the START or STOP buttons. You can adjust the speed or tempo of the rhythm by adjusting this slider. For each rhythm, we want you to adjust the slider so that the tempo or speed is the most appropriate for the given rhythm. Once you have selected what you think is an appropriate tempo for that rhythm, click on the NEXT button to take you to the next rhythm. Take your time in doing this task: try out several different speeds before you decide which is the best for the given rhythm. Remember that there are nine rhythms in total. The counter will tell you how many rhythms you have left in order to complete the experiment.

In addition, participants were asked to complete the *n*–back test and the Trail–Making Test.

***Session 2***

Prior to the second session, the experimenters generated a series of derivative stimuli for each participant. By way of illustration, suppose that for a given rhythm, a participant selected a certain tempo. From this, four other stimuli were derived, one 15% slower, a second 15% faster, a third 30% slower, and a fourth 30% faster. Even slower and even faster stimuli were judged unreasonable by the experimenters. Since each participant had been presented with 9 different rhythms in Session 1, this procedure resulted in 45 rhythmic stimuli for each participant in Session 2.

In the second session, we presented the 45 different rhythms in randomized order and asked the participants to rate their experience of stress and danger. After providing informed consent they were seated in front of a computer screen in a silent and darkened laboratory room. All instructions were given on the screen. Once again, participants wore headphones and were asked to imagine that they were sitting at a campfire somewhere in the savanna while listening to different rhythms. They could close their eyes if they wanted to. Their task was to imagine the scenario as realistically as possible and to evaluate how much stress and danger they would experience in that scenario while listening to each rhythm, using 10-point scales ranging from 1 (*no stress at all* or *safety*), to 10 (*high stress* or *danger*). Participants received the following instructions:

In this experiment you’ll hear a sequence of 45 rhythms. They are based on rhythms that are still common in some regions in Africa. So just imagine you are sitting at a campfire in the savanna, listening to these rhythms. You can close your eyes if you like. For each rhythm we want you to identify how stressed and how safe you would feel in such a situation. Only after listening, try to judge how much you had the experience that you are in a safe situation and that everything is under control and how much stress you felt. You can replay the rhythms by pressing the REPLAY button. The counter will tell you how many rhythms you have left in order to complete the experiment.

### Results and Discussion

The preferred tempi—measured in beats per minute (bpm)—for the nine rhythms presented in Session 1 were *M* = 160.8 bpm (SD = 50.5) for the simple, *M* = 126.0 bpm (SD = 31.8) for the moderate, and *M* = 113.6 bpm (SD = 27.2) for the complex rhythms. These differences are significant, *F*(2,66) = 24.6; *p* < 0.001, η^2^ = 0.43. As the standard deviations show, there was also considerable variation between participants. Participants’ cognitive processing capacity (*M* = 47.5%; SD = 15.1) and speed (*M* = 28.9 s; SD = 25.4) also exhibited large variation. To test our hypothesis that there is an optimum tempo of played rhythms for each listener, depending on the individual’s cognitive processing capacity and speed, we ran a simultaneous regression analysis predicting participants’ mean optimum tempo (averaged across the nine rhythms) by their scores in cognitive processing capacity and speed. Neither cognitive processing capacity (β = 0.05; *p* = 0.80) nor cognitive processing speed (β = –0.09; *p* = 0.59) had an influence on the optimal tempo, *F*(2,34) = 0.21 (*p* = 0.81; *R*^2^ = 0.01). Thus, as expected, the optimum tempo increased as a function of the rhythms’ manipulated information density, but, contrary to our expectations, the optimum tempo did not depend on the listeners’ cognitive processing capacity and speed.

In order to test our main prediction (i.e., that the rhythms of optimum tempo are experienced as least stressful and least dangerous) we ran a contrast analysis. Contrast analysis enables one to test a specific hypothesis directly and is thus more powerful than an analysis of variance (see Rosenthal et al., 2000). According to our hypothesis, we assigned the following contrast weights to the five conditions: preferred tempo minus 30%: 4, preferred tempo minus 15%: –1, preferred tempo: –6, preferred tempo plus 15%: –1, and preferred tempo plus 30%: 4. This *V-shaped contrast* (4, –1, –6, –1, 4) represents the idea of a linear relationship to both sides of an optimum: the more the manipulated tempo deviates from the preferred tempo in either direction the more stress and danger is experienced.

**FIGURE 1:**
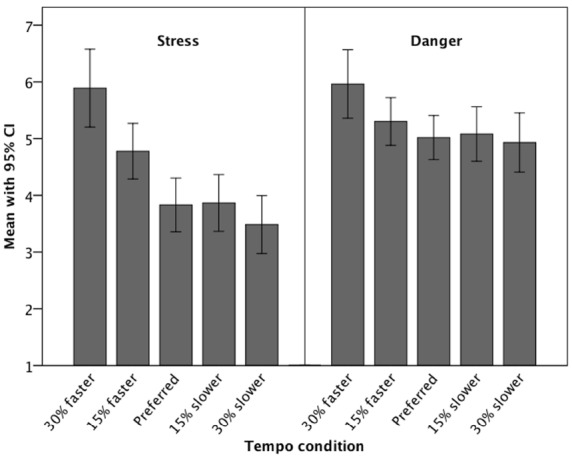
**Means (and 95% confidence intervals) of stress and danger ratings for the five tempo conditions (simple, moderate, and complex rhythms are averaged)**.

The mean stress and danger ratings for the five conditions are shown in Figure 1. For both the stress and the danger ratings, the results are not completely congruent with our predictions. The rhythm variants that were manipulated to have a faster tempo were judged as more stressful and more dangerous that the rhythms with the preferred tempo. The faster the rhythms, the more stressful and dangerous the situation is experienced. The rhythmic variants that were manipulated to have a slower tempo were not judged as more stressful and dangerous, however. Instead, they were judged as eliciting the same levels of stress and danger as the rhythms with the preferred tempo did. Remarkably, though, the stress and danger ratings did not decrease for the slower rhythms as one might expect if complexity and experiences of stress or danger, respectively, were simply in a linear relationship. Contrast analyses revealed a significant fit of the data with the V-shaped contrast, both for the stress ratings (*t* = 5.38; *p* < 0.001; *d* = 0.90) and for the danger ratings (*t* = 3.04; *p* = 0.004; *d* = 0.51). Thus, the results are statistically compatible with the assumption that rhythms that are played slower or faster than the subjectively preferred tempo cause higher levels of experienced stress and danger; but the descriptive statistics show that this only holds for faster tempi while slower tempi led to experiences that are comparable to those in the preferred tempo condition.

## Experiment 2—Experienced Stress and Danger in Situations With Silence, Natural Sounds, and Music

The results of Experiment 1 show that music-like stimuli that vary in their tempo elicit different degrees of experienced stress and danger. Although the data do not perfectly fit our hypotheses they indicate that music-like stimuli may carry information relevant to the processing of environmental safety. If this conjecture is true and the cognitive processing of music is biologically related to the processing of safety-relevant auditory information, we should be able to detect some repercussions of this relationship when comparing real music to other acoustic scenarios. There are two biologically highly relevant scenarios that should be contrasted to music: natural sounds and silence. The study pursued a very simple idea: We had participants sit in front of a computer screen, imagine that they were sitting at a campfire in the savanna, and listen to four different sound scenarios (silence, natural sounds, acoustic instrumental music, and a cappella music). After listening, they were asked to evaluate how stressful and dangerous they had experienced each scenario to be.

### Materials and Methods

In the second experiment, 52 undergraduates participated voluntarily (mean age: *M* = 23.1 years; SD = 4.7; 39 females, 13 males). They all reported unimpaired hearing by self-report. The study was performed in accordance with relevant institutional and national guidelines and regulations (Chemnitz University of Technology, 2002; Deutsche Gesellschaft für Psychologie [German Psychological Society], 2005). Informed consent was obtained from all participants. Anonymity of participants and confidentiality of their data were ensured.

After providing informed consent participants were seated in front of a computer screen in a silent and darkened lab room. All instructions were given on the screen. We asked participants to put on headphones, imagine they were sitting at a campfire somewhere in the African savanna, and listen to four different acoustic scenarios. They could close their eyes if they wanted to. Their task was to imagine the scenarios as realistically as possible and to evaluate how much stress and danger they would experience in each, using 10-point scales ranging from 1 (*no stress at all* or *safety*), to 10 (*high stress* or *danger*).

In the silence condition, participants heard a very low intensity white noise. Quiet noise was employed because true silence rarely occurs in natural acoustic environments. In the savanna condition, participants heard natural savanna sounds from a live recording, consisting of distant animal sounds such as the chirping of crickets. Further, there were two music conditions. Although we had no specific hypothesis about how acoustic *instrumental* music (i.e., only music without vocals) and *a cappella* music (vocals only) would differ, we incorporated these two conditions because chances are that in our prehistoric past, before music instruments were invented, music was only sung (see Kanazawa and Perina, 2012). In the instrumental music condition, participants listened to *Burn* (acoustic instrumental version in Bb minor) by Ellie Goulding; in the vocals only music condition, they listened to *All of me* (voice-only version) by John Legend. Note that we selected these rather slow and calm versions as musical pieces because we sought to analyze if music has the potential to reduce the level of the listeners’ arousal below the level that is present in even silent scenarios. (There was no need to show that arousal can be elevated to a very high degree by very fast, complex, or disliked music because (1) this has already been shown many times before and (2) this did not pertain to the present research question.) All four sound scenarios were edited to 1 min in duration. Participants heard all four scenarios, presented in random order.

### Results and Discussion

**FIGURE 2:**
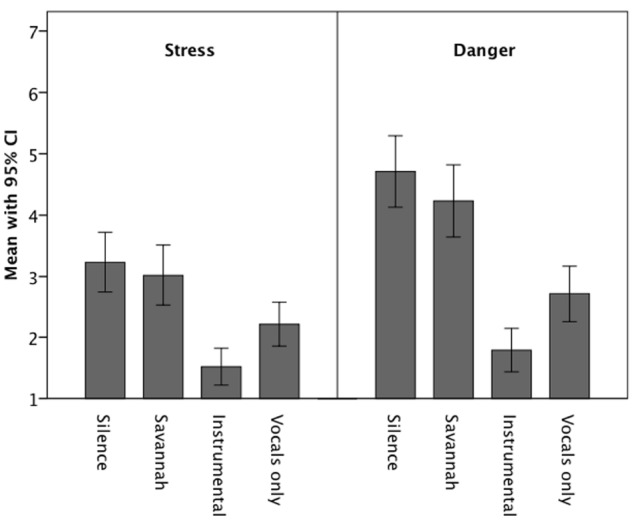
**Means (and 95% confidence intervals) of stress and danger ratings for the four sound scenarios**.

Figure 2 shows the mean stress and danger ratings across the four sound scenarios. Table 1 reports the statistics for all the mean differences. As posited, the two music scenarios elicited less stress and danger than the silence and savanna scenarios, the latter two not exhibiting a significant difference. Thus, compared to when they experienced silent and natural sound scenarios, when participants imagined being in a scenario with music they experienced much less stress and danger. The two music conditions differed significantly, with the instrumental music condition eliciting the lowest stress and danger ratings.

**TABLE 1:** **Bonferroni-corrected ***p***-values and effect sizes for the differences of stress and danger ratings between the four sound scenarios**.

	**Savanna**		**Music only**		**Vocals only**	
	***p***	***d***	***p***	***d***	***p***	***d***
*Stress*						
Silence	> 0.999	0.10	< 0.001	0.84	0.004	0.50
Savanna			< 0.001	0.94	0.009	0.47
Music only					< 0.001	–0.62
*Danger*						
Silence	0.998	0.19	< 0.001	1.33	< 0.001	0.82
Savanna			< 0.001	1.34	< 0.001	0.75
Music only					< 0.001	–0.81

When people are asked to imagine a naturalistic situation in which they are sitting at a campfire in the savanna, relying on auditory information about the safety of the environment—what kind of auditory background would make them feel the most safe and relaxed? In the present study, we used slow and calm music to analyze if that kind of music can reduce the listeners’ arousal level even below that induced by a silent scenario. Data showed that this is the case: Music was the least stressful and dangerous acoustic background, whereas silent scenarios and scenarios with natural sounds elicited stronger experiences of stress and danger. This is remarkable as it shows that silence is not a background sufficient for demonstrating a safe environment where there is no danger, no need to fear anything, no need to act. Instead, people appear to use music as an auditory source of information about the environment. Music might be processed cognitively and evaluated regarding its features—such as its tempo, which we manipulated in the first experiment—to gather information about what is going on “out there.”

The results of Experiment 2 fit the information density hypothesis, which holds that music could provide an acoustic pattern that creates the “illusion” of a safe environment. Music is not simply a constant sine tone or an arbitrary noise but always changes a bit over time, occasionally violating expectations and introducing new elements. These alterations make music a reasonable source of information about the environment on a larger time scale (i.e., beyond a few seconds). On the other hand, music is highly predictable and repetitive. It signals that nothing happens that completely breaks the flux of auditory input.

A more unexpected observation was that the scenario with instrumental music was evaluated as less stressful and dangerous than the scenario with a cappella music. We did not have specific predictions for any difference between the two types of music a priori but we can discuss potential *post hoc* explanations. At least, it is our subjective opinion that the instrumental music was slightly more predictable than the a cappella music. According to the information density hypothesis, higher predictability would equal stronger feelings of safety and relaxation.

## General Discussion

Tens of thousands of years ago, our ancestors did not have comfortable houses where they could lock up, go to sleep, and feel safe. They had to be alert and on their guard against wild animals or human enemies. Yet, being alert and ready to fight or flee all the time is too consumptive of energy and resources. The human body needs time to rest and digest. Especially at night, our ancestors must have relied primarily on their sense of hearing to monitor the environment for possible danger or threats. How that occurred precisely and how humans in general make use of the acoustic information from their environment to evaluate actual danger or safety is a highly interesting though severely under-researched topic (Cross and Watson, 2006).

In this article, we have attempted to take a first and tentative step to address this very basic question. We grounded our research on a tentative hypothesis on how music or music-like sound could influence the human processing of acoustic information pertaining to safety. The results from the two experiments show that (1) there is undoubtedly a relationship between the processing of music and the experience of safety and danger but that (2) our information density hypothesis is not well fitted by the data. According to this hypothesis, safety of an environment would be signaled by auditory input that is neither too scarce nor too complex. Input that is too scarce could be silence or very repetitive and monotonous sounds. Silence represents the absence of auditory input and thus the absence of information about what is going on in the environment. Note that the absence of auditory information could also indicate impaired hearing, which would have been the worst situation for an individual in a prehistoric natural environment. Very repetitive and monotonous sounds also carry little relevant information. Rushing of the wind or water is more comparable to white noise or pink noise; chirping of crickets is very repetitive and monotonous. These sounds would not do much to make an individual feel safe because input with too little newness and change is uninformative input. At the opposite end of the continuum there is auditory input that is too complex. Very fast changing or chaotic sounds might overstrain human cognitive processing capacity and speed, resulting in a failure to adequately process the information that might be covered in the auditory signal. As a consequence, there might be potential information relevant to the individual’s safety that is overlooked in the information flux, resulting in the experience of stress and danger. Therefore, silence or very repetitive and monotonous auditory input as well as very complex auditory input might be expected to evoke feelings of stress or danger. Only input that is, on the one hand, not too repetitive and monotonous and, on the other hand, not too complex or chaotic can be expected to signal a stable and safe environment. Such input is a relevant source of information—because it contains changes and alterations—but is also a signal for a relatively safe environment—because it is highly predictable. Both conditions—not too scarce, not too complex—are fulfilled by music.

Since our hypothesis was not well fitted by the data it is worthwhile considering alternative explanations. We would like to discuss four potential alternatives. (1) Music is known to bear a large influence on the level of physiological arousal, which apparently depends on the energy level of the music’s physical constitution (for an overview, see Huron, 2006). Tempo is the most important physical parameter influencing the listener’s arousal level. Since states of anger, stress, threat, or danger are associated with high levels of arousal, music that leads arousal back to a lower level could, as a result, be associated with the experience of higher safety. Based on that argument, we would expect our data in Experiment 1 to follow a linear contrast: the faster the tempo the more stress and danger is experienced. Given the pattern shown in Figure 1, however, this prediction is just as good or as bad, respectively, as the prediction of a V-shaped relationship. Moreover, regarding Experiment 2, the arousal association idea would predict that the lower the arousal induced through listening to music, the lower the level of experienced stress or danger should be, and, straightforwardly, this level could be lower than the level of arousal caused by natural sound scenarios because these can be associated with actual danger. However, it is not plausible from this hypothesis to suggest that arousal gets lower than when confronted with silence. When taking only the acoustic channel of sensory information processing into account, in a natural environment, silence should be associated with the lowest level of physiological arousal. That is, based on this idea, we could expect music to reduce arousal to the same level as would be associated with silence but not further. The data, however, show that arousal decreased further in the music scenarios. It should be promising for further studies to additionally measure participants’ physiological arousal in different acoustic scenarios. (2) Music is also known to bear a large influence on moods and emotions (see Hunter and Schellenberg, 2010). Parts of our results can be interpreted as effects mediated by mood or emotion. Music can induce moods and emotions through its arousal potential (see above) and valence. In Experiment 1, higher levels of arousal may have intensified potential unpleasant moods/emotions, which, in turn, may have resulted in higher levels of experiences of stress and danger. Yet, following this argument, lower levels of arousal should have weakened potential unpleasant moods/emotions, which was not the case. Moreover, it is quite unlikely that the very simple and artificial MIDI rhythms actually induced any specific moods or emotions in our participants. By contrast, in Experiment 2, it is quite likely that the music induced pleasant moods or emotions that may have masked the unpleasant experiences of stress and danger. (3) Music could provide a perceptual analogy with threatening sounds such as approaching footsteps. Specifically, the perception of rapid footsteps could signal aggressive predators or hostile conspecifics approaching rapidly with ambiguous intentions^1^. As a result, slow music could signal that no such threat is to be feared. This idea would also result in a linear contrast and is thus also not well fitted by the data. Data from Experiment 2 do not support the idea of perceptual analogy, either. We would expect that the level of experienced stress or danger could get as low as in silent or natural sound scenarios, namely, when the musical sound does not mirror any kind of threatening event. We would not expect, however, music scenarios to elicit levels of experienced stress or danger below those resulting from a silent scenario. (4) If an individual is in danger or is monitoring for danger, he or she is less likely to be making music. Thus, engagement in making music suggests that no danger is present^1^. As a result, if a listener hears someone else making music, that listener can assume that the music maker feels safe enough to devote attention to music making and not to a dangerous situation or to scanning the environment for dangers. Based on this idea, regarding Experiment 1, no specific differences between the five tempo conditions would be expected. However, the idea would be congruent with the data from Experiment 2 because it would predict that music scenarios are experienced as less stressful and dangerous than silent or natural sound scenarios. Natural sounds could mask potentially safety-relevant acoustic information. Silence, too, is not necessarily indicative of a safe environment since approaching danger can be very quiet. Hearing somebody making music would signal that this person is not in danger and not monitoring for danger, which can also be recognized with eyes closed, for instance.

The data from Experiment 1 show a large variation in preferred tempi. We had expected that cognitive information-processing capacity and speed would explain this variance, but this was not the case. It therefore remains an open question what causes different listeners to feel comfortable with different listening tempi. Music education variables might play a role, as might other personality characteristics that were not recorded in the present study.

We consider our studies a cautious first step that certainly has its limitations. First, it is of course hard to induce the experience of being in a naturalistic real-world situation when actually being in a lab, let alone our intention to induce the experience of stress and danger by the scenarios used. Yet, data show that the manipulations in both experiments indeed led to variance in the experience of stress and danger. Notably, that variance does not seem to be simply a result of demand characteristics: We asked the majority of participants after Experiment 1 if they had recognized that they had just listened to tempo variants of the same rhythms, which they all denied. The rhythms were simply too unfamiliar to them. Second, the effectiveness of the procedure we used depends on how well participants are able to produce mental imagery. It is known that there are individual differences in mental imagery ability (e.g., Marks, 1973; Kosslyn et al., 1984) and, more specifically, in how mental imagery evokes emotions (Holmes and Mathews, 2005, 2010). These differences should be addressed in follow-up studies and analyzed in relation to the stress and danger ratings. In addition, future studies on experiences of stress and danger may employ designs without mental imagery. Having participants complete a stressful and/or dangerous task or computer game would induce stress and danger more naturalistically; and experimentally manipulated background music could be investigated regarding its potential to mask or attenuate nascent feelings of stress and danger. A third limitation is the uneven gender distribution in both experiments, which simply mirrors the gender distribution of the local university’s psychology students. We have not reported separate analyses, however, because there were too few female participants, which would have resulted in low statistical power. Despite these potential limitations, we believe that our data can help shed a little more light on the potential role of musical sounds in the processing of safety-relevant information in prehistoric environments.

### Conflict of Interest Statement

The authors declare that the research was conducted in the absence of any commercial or financial relationships that could be construed as a potential conflict of interest.

## Footnotes

1 We thank a reviewer of an early version of our manuscript for directing us to this potential alternative explanation.
